# ST3Gal IV Mediates the Growth and Proliferation of Cervical Cancer Cells *In Vitro* and *In Vivo* Via the Notch/p21/CDKs Pathway

**DOI:** 10.3389/fonc.2020.540332

**Published:** 2021-02-01

**Authors:** Yinshuang Wu, Xixi Chen, Weijie Dong, Zhongyang Xu, Yuli Jian, Chunyan Xu, Lin Zhang, Anwen Wei, Xiao Yu, Shidan Wang, Yue Wang, Gang Liu, Xiaoxin Sun, Shujing Wang

**Affiliations:** ^1^ Department of Biochemistry and Molecular Biology, College of Basic Medical Sciences, Institute of Glycobiology, Dalian Medical University, Dalian, China; ^2^ Department of Biological Sciences, School of Life Science and Medicine, Dalian University of Technology, Panjin, China; ^3^ Department of Gynaecology, Jiaxing University Affiliated Women and Children Hospital, Jiaxing, China; ^4^ Department of Pathology, College of Basic Medical Sciences, Dalian Medical University, Dalian, China

**Keywords:** ST3Gal IV, cervical cancer, α2, 3-sialylation, cell proliferation, Notch signaling pathway

## Abstract

ST3Gal IV is one of the principal sialyltransferases responsible for the biosynthesis of α2, 3-sialic acid to the termini *N*-glycans or *O-*glycans of glycoproteins and glycolipids. It has been reported that ST3Gal IV expression is associated with gastric carcinoma, pancreatic adenocarcinoma and breast cancer. While the expression and functions of ST3Gal IV in cervical cancer are still poorly understood. In this study, we found that ST3Gal IV was downregulated in human cervical cancer tissues compared to normal cervix tissues, and ST3Gal IV expression was negatively associated with the pathological grade of cervical cancer. ST3Gal IV upregulation inhibited the growth and proliferation of cervical cancer HeLa and SiHa cells *in vitro* and *in vivo*. Furthermore, ST3Gal IV overexpression enhanced the expression of several Notch pathway components such as Jagged1, Notch1, Hes1 and Hey1, while cell cycle protein expression like Cyclin D1, Cyclin E1, CDK2 and CDK4 were decreased. These results indicate that expression of ST3Gal IV is reduced in cervical cancer and plays a negative role in cell proliferation *via* Notch/p21/CDKs signaling pathway. Thus, sialyltransferase ST3Gal IV might be a target for the diagnosis and therapy of cervical cancer.

## Introduction

Cervical cancer is one of the leading causes of cancer death among females, and its death rate ranks second among cancers in less developed countries (1). It has been demonstrated that cervical cancer is caused by human papillomavirus (HPV) infection (2). Significant advances have been made in cervical cancer screening and treatment. The development of HPV and its widespread adoption may contribute to reduced incidence of the disease. However, the incidence and mortality rates are still high due to the difficulties in achieving widespread compliance with HPV vaccination (3). In addition, HPV vaccines cannot protect against all the types of HPV that cause cervical cancer (1). Therefore, we focus more on the mechanism of cervical cancer occurrence and development, and try to find more ways to overcome cervical cancer.

Sialylation is one of the most common glycosylation processes which adds sialic acids to the terminal of glycoconjugates. Current research suggests that sialylation is involved in various cellular functions such as cell adhesion and signal transduction. Sialyltransferases (STs) include 20 members and are divided into four families: ST3Gal I-VI, ST6Gal I-II, ST6GalNAc I-VI, and ST8Sia I-VI (4). These sialyltransferases are responsible for the transfer of sialic acid and participate in the post-translational modifications of proteins. There is increasing evidence that abnormal expression levels of sialyltransferases are related to tumor proliferation, invasion, metastasis and drug resistance (5–7).

ST3Gal IV is one of the major enzymes responsible for the formation of α2, 3-sialylated *N*-glycan or *O*-glycan (8, 9). It has been reported that the expression of ST3Gal IV is associated with various tumors such as gastric cancer, head and neck squamous cell carcinoma (HNSCC) (10) and renal cell carcinoma. Pérez-Garay et al. demonstrated that ST3Gal IV was highly expressed in pancreatic adenocarcinoma tissues and enhanced the migration and metastasis of pancreatic adenocarcinoma cells (11). Saito et al. found that the mRNA level of ST3Gal IV in primary renal cell carcinoma tissues was decreased compared with non-tumor kidney tissues, and that the down-regulated ST3Gal IV was considered to be one of the factors related to the malignant progression of renal cell carcinoma (12). However, whether ST3Gal IV is involved in the proliferation of cervical cancer cells and the mechanisms behind these observations remain poorly understood.

In this study, combined with cervical cancer tissue chips and clinical baseline data analysis, we found that the expression level of ST3Gal IV in cervical cancer tissues was lower than that in adjacent tissues, and was negatively correlated with the malignancy of the tumor. The overexpression of ST3Gal IV in cervical cancer cells inhibited the proliferation, migration and invasion capability of SiHa and HeLa cells. ST3Gal IV overexpression inhibited the proliferation and tumorigenesis of HeLa cells in nude mice. In addition, the expression levels of Jagged1, Notch1, Hes1 and Hey1 in Notch signaling pathway were increased upon ST3Gal IV upregulation, while p21, CyclinD1, CyclinE1, CDK2 and CDK4 protein levels were decreased. Moreover, Notch inhibitor DAPT restored the expression levels of above-mentioned proteins in cells with overexpression of ST3Gal IV. In conclusion, ST3Gal IV expression inhibits the growth and proliferation of cervical cancer cells *in vitro* and *in vivo* through the Notch1/p21/CDKs signaling pathway.

## Materials and Methods

### Cell Culture

Cervical carcinoma cell lines SiHa and HeLa were purchased from the Cell Bank of Chinese Academy of Sciences (Shanghai, China) and maintained in Dulbecco’s modified Eagle’s medium (DMEM) (Gibco, Novato, CA, United States). The medium contains 10% fetal bovine serum (Gibco, Novato, CA, United States), 100 U/ml penicillin, and 100 U/ml streptomycin (Beyotime, China). Then the cell lines were cultured in cell incubator at 37°C with 5% CO_2_.

### Immunohistochemistry

Tumor tissues from xenograft model and commercial tissue arrays (Superbiotek, Shanghai, China) were dewaxed in dimethylbenzene and hydrated in gradient alcohol. After antigen retrieval and blocking, primary antibody (1:80; Proteintech, 13546-1-AP) against ST3Gal IV was incubated with the tissue samples overnight at 4°C. Then the biotinylated secondary antibody was used to combine with the primary antibodies and incubated for 30 min at 37°C. Diaminobenzidine (DAB) (ZSGB-BIO, Beijing, China) was used as a developer, and hematoxylin was used for counterstaining. Two observers reviewed the immunohistochemical staining results independently and assessed the staining intensity and extent by German semi-quantitative scoring system (13). The cut-off value for differentiating between final positive and negative immunostaining was set at 4 by using the receiver operating characteristic (ROC) curve analysis (14, 15). Score of 0 to 3 points was considered negative for protein expression and score of 4 points or greater was considered positive which showed high protein expression.

### Cell Transfection

HeLa and SiHa cells were treated with a mixture of recombinant pcDNA3.1/ST3Gal IV vector or a control vector and Lipofectamine 2000 (Invitrogen, Carlsbad, CA, USA) depending on the manufacturer’s instructions to increasing the expression of ST3Gal IV or as a control group. After 48 h, 600 µg/ml, 800 µg/ml of G418 (Sigma-Aldrich, Darmstadt, Germany) were used to select stably transfected HeLa and SiHa cells respectively. The expression of ST3Gal IV was confirmed by RT-PCR, Western blot and Lectin blot.

### Real-Time PCR

Total RNA was extracted from normal, control and transfected HeLa, SiHa cells by TRIzol reagent (Life Technologies, Carlsbad, CA, USA) and employed to synthesize cDNA using a PrimeScript RT Reagent Kit (Takara, Dalian, China). Subsequently, cDNA mixed with qPCR SuperMix (Takara, Otsu, Japan) and GAPDH or ST3Gal IV primer (GenePharma) then reacted at 94°C for 3 min, 94°C for 5 s and 60°C for 34 s with 40 cycles. The expression levels were analyzed using the 2^−ΔΔCT^ method and the GAPDH was used as an internal control.

### Western Blot Assay

Proteins were extracted from cells and determined by BCA kit (Beyotime). Equal amounts of protein were separated by 10% SDS-PAGE (sodium dodecyl sulfate polyacrylamide gel electrophoresis) and transferred onto PVDF membranes (Pall Corporation, New York, NY, USA). Then we blocked the membranes with 5% skim milk in TBST at room temperature for 2 h. The membranes were incubated with primary antibodies of ST3Gal IV (1:1000, proteintech, 13546-1-AP), Jagged1 (1:750, Elabscience, ENK5401), Notch (1:750, proteintech, 20687-1-AP), Hes1 (1:1000, Elabscience, EAP2709), Hey1 (1:1250, proteintech, 19929-1-AP), p21 (1:1000, Bioworld, BS6501), p53 (1:1000, Bioworld, BS3156), Cyclin D1 (1:1000, Affinity biosciences lnc, DF6386), Cyclin E1 (1:1000, Bioworld, BZ00342), CDK2(1:1000, Bioworld, BS1050), CDK4 (1:1000, Bioworld, BS6462), GAPDH (1:4000, proteintech, 10494-1-AP) overnight at 4°C. Subsequently, these membranes were incubated with secondary antibody (1:10,000, ZSGB-BIO, ZB-2301) for 2 h at room temperature. The protein bands on the membranes were visualized by ECL kit (Advansta, Menlo Park, CA, USA) and conducted with the Image Lab software (Bio-Rad, Hercules, CA, USA).

### Lectin Blot Analysis

Similar to the Western blot assay, about 30 μg of protein was loaded onto two 10% SDS-polyacrylamide gels while one was subjected to Coomassie Brilliant Blue (CBB) staining and the other was transferred onto PVDF membranes. The membrane was blocked in 5% skim milk at room temperature for 2 h and incubated with Maackia Amurensis Lectin II (MAL II, 1:1000, Vector Laboratories, B-1265) or biotin-labeled Lectin Sambucus nigra agglutinin (SNA, 1:2000, Vector) for 2 h at 37°C. Then the membrane was incubated with horseradish peroxidase streptavidin (1:1000, ZSGB-BIO) for an hour at room temperature. Image Lab software (Bio-Rad) was used for detection and Gel-Pro software worked as analyzer.

### Purification of *N*-Glycans from Cell Membrane Proteins

Cell membrane proteins were extracted using a CelLytic MEM Protein Extraction kit (Sigma) according to the manufacturer’s instruction. The proteins (200 μg per group) were mixed with 50 mM NH_4_HCO_3_ and 50 mM dl-dithiothreitol (DTT) and reduced at 56°C for 30 min. The alkylation was carried out by adding 50 mM iodoacetamide followed by incubation in dark at room temperature for 1 h. The mixtures were then treated with trypsin (2 μg) at 37°C for 16 h, and inactivation at 95°C for 5 min. The sample was finally incubated with 3 mU peptide *N*-glycanase F (PNGase F) (Takara) at 37°C overnight and vacuum dried. The released *N*-glycans were purified using an Oasis MCX cartridge (30 mg/ml; Waters) and lyophilized.

Permethylation of the freeze-dried *N*-glycans was performed according to the method described previously (16) with some minor changes. Dimethyl sulfoxide (DMSO) containing 1% v/v distilled water (50 μl) was added to the dried glycan sample under alkaline conditions with powdered sodium hydroxide. The mixtures were treated with Methyl iodide (50 μl) at room temperature for 30 min with vigorous shaking. Distilled water (1 ml) was slowly added to the reaction mixture, and then the mixture was applied to a Sep-Pak Vac 18 cartridge (50 mg/ml; Waters). Finally, the permethylated glycans were eluted with 80% v/v acetonitrile in distilled water and evaporated to dryness.

### Mass Spectrometry (MS)

MS Spectra of the permethylated glycans were acquired using a matrix-assisted laser desorption/ionization-time of flight/time of flight (MALDI-TOF/TOF) MS (New ultrafleXtreme, Bruker Daltonik). Ions were generated by signal averaging over 4,000 laser shots from a Smartbeam-II Nd : YAG laser operating at 355 nm and a repetition rate of 2 kHz. All spectra were obtained in the reflectron mode with delayed extraction of 200 ns. For sample preparation, 0.5 μl of 2, 5-dihydroxybenzoic acid (DHB, 10 mg/ml) in 30% ethanol was spotted onto a target plate (MTP 384 target plate ground steel, Bruker Daltonik). After dried, an aliquot (0.5 μl) of the glycan solution was spotted onto the DHB crystal and dried. Mass spectra were obtained from Na^+^ adductions.

### Cell Survival Assays by Cell Counting Kit-8 and Cell Colony Formation Assay

Cells were plated in 96-well plates at a density of 4 × 10^3^ cells per well in triplicate and 10 μl of CCK-8 reagent was added into each well and incubated at 37°C with 5% CO_2_ for an hour. Then the microplate reader (Thermo Fisher Scientific) was used to measure the absorbance at 450 nm. Moreover, 1 × 10^3^ cells per well were seeded in 6-well plates and incubated for two weeks. Subsequently, cells were fixed with methanol and stained with 0.1% crystal violet. The cell clusters comprised more than fifty cells.

### Flow Cytometry

Cells were gathered as single cell suspension and fixed with 70% alcohol at 4°C overnight. Then centrifuged for 5 min at 1,000 rpm and washed two times by cold PBS liquid to get rid of alcohol. Cells were re-suspended in PBS containing 50 μg/ml RNase and 50 μg/ml PI (propidium iodide) and incubated for 30 min at 4°C in the dark. RNase is used to decompose RNA in cells and PI for the staining of DNA. Subsequently, flow cytometer (FACSCalibur, BD, USA) with CELLquest pro software analyzed the cell cycle distribution by detecting the fluorescence intensity of PI.

### EdU Assay

EdU (5-ethynyl-2′-deoxyuridine) as a nucleoside analog of thymidine could involve in the DNA synthesis then measure the cell proliferation ability (17) and reflect cell cycle distribution. Cells were plated on slips at appropriate density for 12 h and treated with EdU kit (Invitrogen, USA) according to the manual. The fluorescence was observed by confocal laser-scanning microscope (BD, Biosciences).

### Xenograft Model

Nude mice aged 4 to 6 weeks were obtained from the Animal Experiment Center of Dalian Medical University and divided into a HeLa group and two HeLa/ST3Gal IV groups randomly. 2 × 10^7^ cells were injected into the right dorsal flank of each mouse and the tumor diameters were measured by vernier caliper every 5 days a week later. After a month, all mice were killed and the weights of tumors were determined, and the volumes were calculated by the formula: 1/2 (length × width^2^) (18).

### Statistical Analysis

Data were presented as mean ± SD and analyzed with SPSS 20.0 (SPSS Inc., USA) and each assay was performed at least three times independently. The differences between two groups were determined by Student’s t-test and among three or four groups were analyzed by one-way ANOVA. *P* < 0.05 was considered to be statistically significant.

## Results

### ST3Gal IV Expression Is Negatively Related to the Malignant Degree of Cervical Cancer Tissues

**Figure 1:**
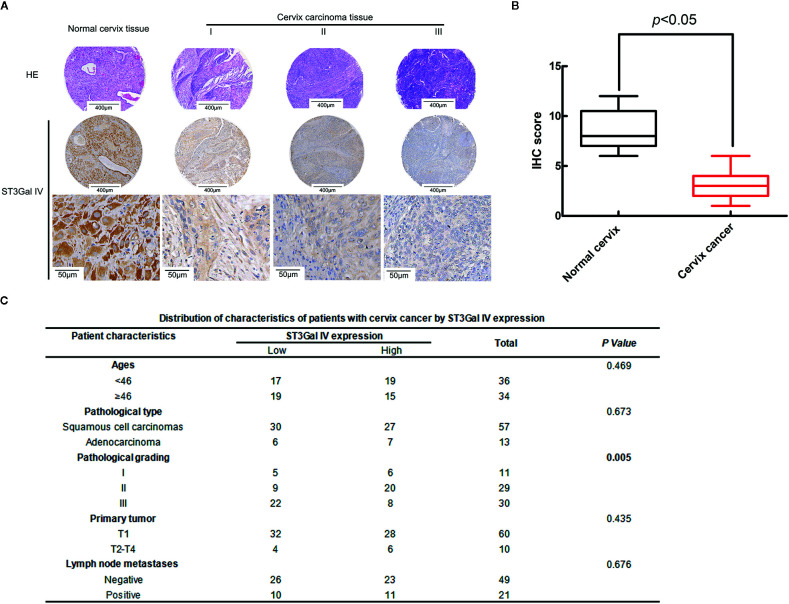
ST3Gal IV downregulation was associated with the malignant degree of cervical cancer tissues. **(A)** IHC analysis of ST3Gal IV expression in normal cervix tissues and different grades of cervical cancer tissues. (The first and second rows: magnification, ×4, scale bars, 400 μm; the third row: magnification, ×40, scale bars, 50 μm). **(B)** Boxplot of IHC scores shown ST3Gal IV protein expression levels in normal cervix tissues and cervix cancer tissues, *p* < 0.05. **(C)** Pearson’s chi-squared test identified a significant correlation between ST3Gal IV expression with cancer tissue pathologic grade, *p* = 0.005 (normal tissue did not participate in statistics).

To analyze the expression levels of ST3Gal IV in the development of cervical cancer, 75 cases of cervical cancer tissue microarray were evaluated by immunohistochemistry (IHC). As shown in **Figure 1A**, the expression of ST3Gal IV decreased with the increase of the malignancy of cervical cancer. Box plots of IHC scores for ST3Gal IV expression also showed that ST3Gal IV expression was lower in cervical cancer tissue compared to normal cervical tissue (**Figure 1B**). According to the IHC score, 70 cervical cancer cases were subdivided into a “low ST3Gal IV expression” group containing 36 samples (score of 0 to 3) and a “high ST3Gal IV expression” group containing 34 samples. Five cases of normal tissues did not participate in statistics. The association between ST3Gal IV expression and patient ages, pathological types, pathological grading, primary tumor stages and lymph node metastasis was assessed by Pearson’s chi-squared test (**Figure 1C**). These results suggest that ST3Gal IV expression was negatively correlated with pathological grading of cervical cancer tissues (*p* = 0.005).

### Establishment of Stable ST3Gal IV-Upregulated Cervical Cancer Cell Lines

**Figure 2:**
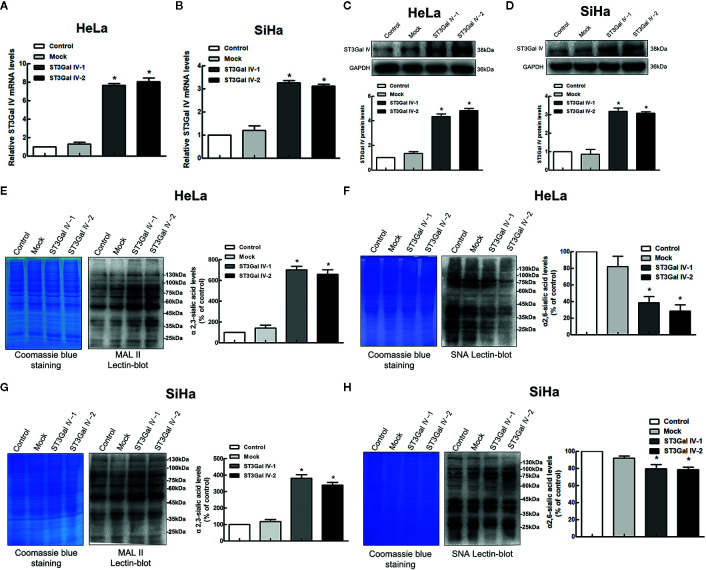
ST3Gal IV upregulation increased α2, 3-linked sialic acid levels in HeLa and SiHa cells. **(A, B)** The mRNA level of ST3Gal IV was analyzed by real-time PCR. **(C, D)** ST3Gal IV protein expression was determined by Western blot. **(E–H)** α2,3 and α2,6-linked sialic acid levels in HeLa and SiHa cells were measured by Lectin blot analysis. (The left panel shows total protein (Coomassie) and the right panel shows the Lectin blot.) The Western blot and Lectin blot results shown in the figure are representative results. All data are from three independent experiments and shown as mean ± SD. * on behalf of *p* < 0.05.

To further explore whether ST3Gal IV is associated with the development of cervical cancer, the expression of ST3Gal IV was up-regulated in two typical cervical cancer cell lines, HeLa and SiHa cells. Real-time PCR results showed that the mRNA levels of ST3Gal IV were increased significantly compared to the control and mock cells (**Figures 2A, B**). Meanwhile, ST3Gal IV protein expression was increased (**Figures 2C, D**). Furthermore, Lectin blot analysis (**Figures 2E–H**) showed that ST3Gal IV overexpression enhanced the expression of α2, 3-linked sialic acid and decreased the expression levels of α2, 6-linked sialic acid in HeLa and SiHa cells. These results suggest that ST3Gal IV can be stably overexpressed in HeLa and SiHa cells by transfecting the overexpression vector.

### MALDI-TOF MS Analysis for *N*-glycan Profiling of Cervical Cancer Cell Lines

**Figure 3:**
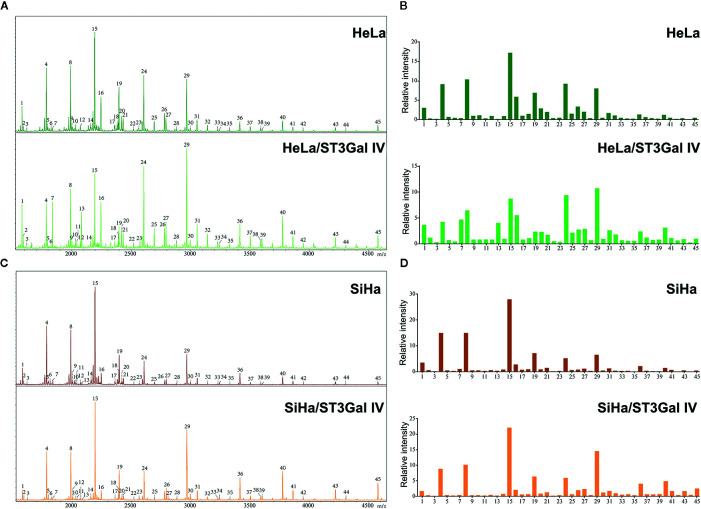
ST3Gal IV overexpression increased sialylated glycans in cervical cancer cell lines. **(A, C)** *N*-glycan from HeLa, HeLa/ST3Gal IV, SiHa, and SiHa/ST3Gal IV cells were permethylated and analyzed by MALDI-TOF MS/MS. **(B, D)** The relative intensities of the observed glycan signals (1–45) in these four cervical cancer cell lines. The results shown in the figure are representative results.

To determine whether the profiles of *N*-glycan are altered in cervical cancer cells after overexpression of ST3Gal IV, we compared *N*-linked glycans on cell membranes produced by HeLa, HeLa/ST3Gal IV, SiHa, and SiHa/ST3Gal IV cells. The MS spectrum and the monosaccharide composition for each signal were summarized in **Figure 3** and **Table 1**, respectively. In total, 45 kinds of glycoforms were detected and relatively quantified in these four cell lines. *N*-glycan compositions with the most intense signal detected in HeLa cells was peak 15, followed by 8, 24, 4, 29, 19, 16 (in the order of decreasing signal strength), while in HeLa/ST3Gal IV cells was peak 29, followed by 24, 15, 8, 16, 7, 4. Among these *N*-linked glycans, peak 29 (*m/z* = 2966.18) [(Hex)_2_(HexNAc)_2_(Fuc)_1_(NeuAc)_2_+(Man)_3_(GlcNAc)_2_] and peak 24 (*m/z* = 2605.42) [(Hex)_2_(HexNAc)_2_(Fuc)_1_(NeuAc)_1_+(Man)_3_(GlcNAc)_2_] representing sialylated glycans were significantly increased in HeLa/ST3Gal IV cells. In addition, peak 4, 8, 15, 19 representing high-mannose type glycans were dramatically decreased in HeLa/ST3Gal IV cells. Similar results were also obtained in SiHa and SiHa/ST3Gal IV cells. These data indicated that overexpression of ST3Gal IV increased sialylated glycans on the membrane proteins of cervical cancer cells.

**Table 1:** Summary of *N*-linked glycans released from HeLa, HeLa/ST3GalIV and SiHa, SiHa/ST3GalIV cell lines in MALDI-TOF-MS.

Peak	Observed *m/z*				Calculated *m/z^a^* ^)^	Chemical composition^b）^
No.	HeLa	HeLa/ST3GalⅣ	SiHa	SiHa/ST3GalⅣ		
1	1579.48	1579.65	1579.49	1579.60	1579.78	(Hex)_2_ + (Man)_3_(GlcNAc)_2_
2	1590.50	1590.67	1590.51	1590.63	1590.80	(HexNAc)_1_ (Fuc)_1_ + (Man)_3_(GlcNAc)_2_
3	1620.51	1620.68	1620.55	1620.63	1620.81	(Hex)_1_ (HexNAc)_1_ + (Man)_3_(GlcNAc)_2_
4	1783.62	1783.82	1783.65	1783.77	1783.88	(Hex)_3_ + (Man)_3_(GlcNAc)_2_
5	1794.65	1794.84	1794.68	1794.78	1794.90	(Hex)_1 _(HexNAc)_1_ (Fuc)_1_ + (Man)_3_(GlcNAc)_2_
6	1824.67	1824.87	1824.69	1824.82	1824.91	(Hex)_2_ (HexNAc)_1_ + (Man)_3_(GlcNAc)_2_
7	1835.69	1835.89	1835.73	1835.84	1835.92	(HexNAc)_2_ (Fuc)_1_ + (Man)_3_(GlcNAc)_2_
8	1987.81	1988.03	1987.85	1987.97	1987.98	(Hex)_4_ + (Man)_3_(GlcNAc)_2_
9	1998.83	1999.04	1998.89	1998.98	1999.00	(Hex)_2 _(HexNAc)_1_ (Fuc)_1 _+ (Man)_3_(GlcNAc)_2_
10	2028.85	2029.08	2028.90	2029.03	2029.01	(Hex)_3_ (HexNAc)_1_ + (Man)_3_(GlcNAc)_2_
11	2039.87	2040.09	2039.93	2040.05	2040.02	(Hex)_1_ (HexNAc)_2_ (Fuc)_1_ + (Man)_3_(GlcNAc)_2_
12	2069.89	2070.11	2069.94	2070.07	2070.03	(Hex)_2_ (HexNAc)_2_ + (Man)_3_(GlcNAc)_2_
13	2080.93	2081.14	2080.96	2081.06	2081.05	(HexNAc)_3 _(Fuc)_1_ + (Man)_3_(GlcNAc)_2_
14	2155.96	2156.19	2156.00	2156.13	2156.07	(Hex)_1 _(HexNAc)_1_ (Fuc)_1_ (NeuAc)_1_ + (Man)_3_(GlcNAc)_2_
15	2191.98	2192.22	2192.03	2192.16	2192.08	(Hex)_5_ + (Man)_3_(GlcNAc)_2_
16	2244.04	2244.28	2244.11	2244.23	2244.12	(Hex)_2 _(HexNAc)_2 _(Fuc)_1 _+ (Man)_3_(GlcNAc)_2_
17	2360.09	2360.35	2360.15	2360.27	2360.17	(Hex)_2_ (HexNAc)_1_ (Fuc)_1 _(NeuAc)_1_ + (Man)_3_(GlcNAc)_2_
18	2390.10	2390.36	2390.17	2390.29	2390.18	(Hex)_3_ (HexNAc)_1_ (NeuAc)_1_ + (Man)_3_(GlcNAc)_2_
19	2396.10	2396.37	2396.16	2396.30	2396.18	(Hex)_6_ + (Man)_3_(GlcNAc)_2_
20	2418.13	2418.39	2418.19	2418.32	2418.21	(Hex)_2 _(HexNAc)_2_ (Fuc)_2_ + (Man)_3_(GlcNAc)_2_
21	2431.13	2431.39	2431.19	2431.32	2431.21	(Hex)_2_ (HexNAc)_2_ (NeuAc)_1_ + (Man)_3_(GlcNAc)_2_
22	2519.16	2519.42	2519.23	2519.33	2519.26	(Hex)_3_ (HexNAc)_3_ + (Man)_3_(GlcNAc)_2_
23	2564.14	2564.41	2564.20	2564.34	2564.27	(Hex)_3_ (HexNAc)_1 _(Fuc)_1_ (NeuAc)_1_ + (Man)_3_(GlcNAc)_2_
24	2605.15	2605.42	2605.21	2605.35	2605.30	(Hex)_2_ (HexNAc)_2_ (Fuc)_1_ (NeuAc)_1_ + (Man)_3_(GlcNAc)_2_
25	2693.14	2693.41	2693.21	2693.34	2693.35	(Hex)_3_ (HexNAc)_3 _(Fuc)_1_ + (Man)_3_(GlcNAc)_2_
26	2779.07	2779.37	2779.15	2779.29	2779.39	(Hex)_2 _(HexNAc)_2_ (Fuc)_2_ (NeuAc)_1_ + (Man)_3_(GlcNAc)_2_
27	2792.07	2792.35	2792.13	2792.27	2792.38	(Hex)_2_ (HexNAc)_2_ (NeuAc)_2_ + (Man)_3_(GlcNAc)_2_
28	2879.97	2880.29	2880.06	2880.21	2880.44	(Hex)_3_ (HexNAc)_3_ (NeuAc)_1_ + (Man)_3_(GlcNAc)_2_
29	2965.87	2966.18	2965.94	2966.10	2966.47	(Hex)_2_ (HexNAc)_2_ (Fuc)_1 _(NeuAc)_2 _+ (Man)_3_(GlcNAc)_2_
30	2995.83	2996.14	2995.91	2996.06	2996.48	(Hex)_3_ (HexNAc)_2_ (NeuAc)_2_ + (Man)_3_(GlcNAc)_2_
31	3053.74	3054.06	3053.81	3053.98	3054.52	(Hex)_3_ (HexNAc)_3 _(Fuc)_1_ (NeuAc)_1_ + (Man)_3_(GlcNAc)_2_
32	3141.56	3141.89	3141.66	3141.81	3142.58	(Hex)_4_ (HexNAc)_4 _(Fuc)_1_ + (Man)_3_(GlcNAc)_2_
33	3227.35	3227.68	3227.42	3227.60	3228.61	(Hex)_3_ (HexNAc)_3_ (Fuc)_2_ (NeuAc)_1_ + (Man)_3_(GlcNAc)_2_
34	3240.32	3240.65	3240.39	3240.57	3239.63	(Hex)_1 _(HexNAc)_4_ (Fuc)_3_ (NeuAc)_1_ + (Man)_3_(GlcNAc)_2_
35	3328.06	3328.40	3328.13	3328.31	3327.65	(Hex)_2_ (HexNAc)_2_ (Fuc)_1 _(NeuAc)_3 _+ (Man)_3_(GlcNAc)_2_
36	3413.77	3414.11	3413.84	3414.02	3413.72	(Hex)_1_ (HexNAc)_4_ (Fuc)_4 _(NeuAc)_1_ + (Man)_3_(GlcNAc)_2_
37	3501.44	3501.80	3501.52	3501.70	3501.74	(Hex)_2 _(HexNAc)_2_ (Fuc)_2 _(NeuAc)_3_ + (Man)_3_(GlcNAc)_2_
38	3587.08	3587.43	3587.12	3587.32	3587.81	(Hex)_1_ (HexNAc)_4_ (Fuc)_5_ (NeuAc)_1_ + (Man)_3_(GlcNAc)_2_
39	3600.02	3600.36	3600.10	3600.26	3600.80	(Hex)_1_ (HexNAc)_4_ (Fuc)_3_(NeuAc)_2_ + (Man)_3_(GlcNAc)_2_
40	3773.09	3773.47	3773.18	3773.38	3772.30	(Hex)_2_ (HexNAc)_6_ (NeuAc)_2_ + (Man)_3_(GlcNAc)_2_
41	3860.57	3860.97	3860.66	3860.87	3860.94	(Hex)_3_ (HexNAc)_7_ (NeuAc)_1_ + (Man)_3_(GlcNAc)_2_
42	3948.00	3948.41	3948.10	3948.31	3948.99	(Hex)_4_ (HexNAc)_8_ + (Man)_3_(GlcNAc)_2_
43	4218.84	4219.30	4218.95	4219.19	4219.10	(Hex)_7_ (HexNAc)_3 _(Fuc)_3_ (NeuAc)_1_ + (Man)_3_(GlcNAc)_2_
44	4306.10	4306.51	4306.21	4306.40	4307.15	(Hex)_8_ (HexNAc)_4_ (Fuc)_3_ + (Man)_3_(GlcNAc)_2_
45	4576.21	4576.68	4576.34	4576.57	4576.26	(Hex)_6_ (HexNAc)_3_ (NeuAc)_4_ + (Man)_3_(GlcNAc)_2_

a) The glycans were calculated as permethylated glycan and [M + Na]^+^.

b) Monosaccharide compositions were determined by database searching using GlycoMod (http://www.expasy.ch/tools/glycomod/).

Monosaccharides are indicated as follows: Hex, hexose; HexNAc, N-acetyl hexosamine; Fuc, fucose; NeuAc, N-acetyl neuraminic acid; Man, mannose.

### ST3Gal IV Overexpression Inhibits the Proliferation and Colony Formation Ability of HeLa and SiHa Cells *In Vitro*

**Figure 4:**
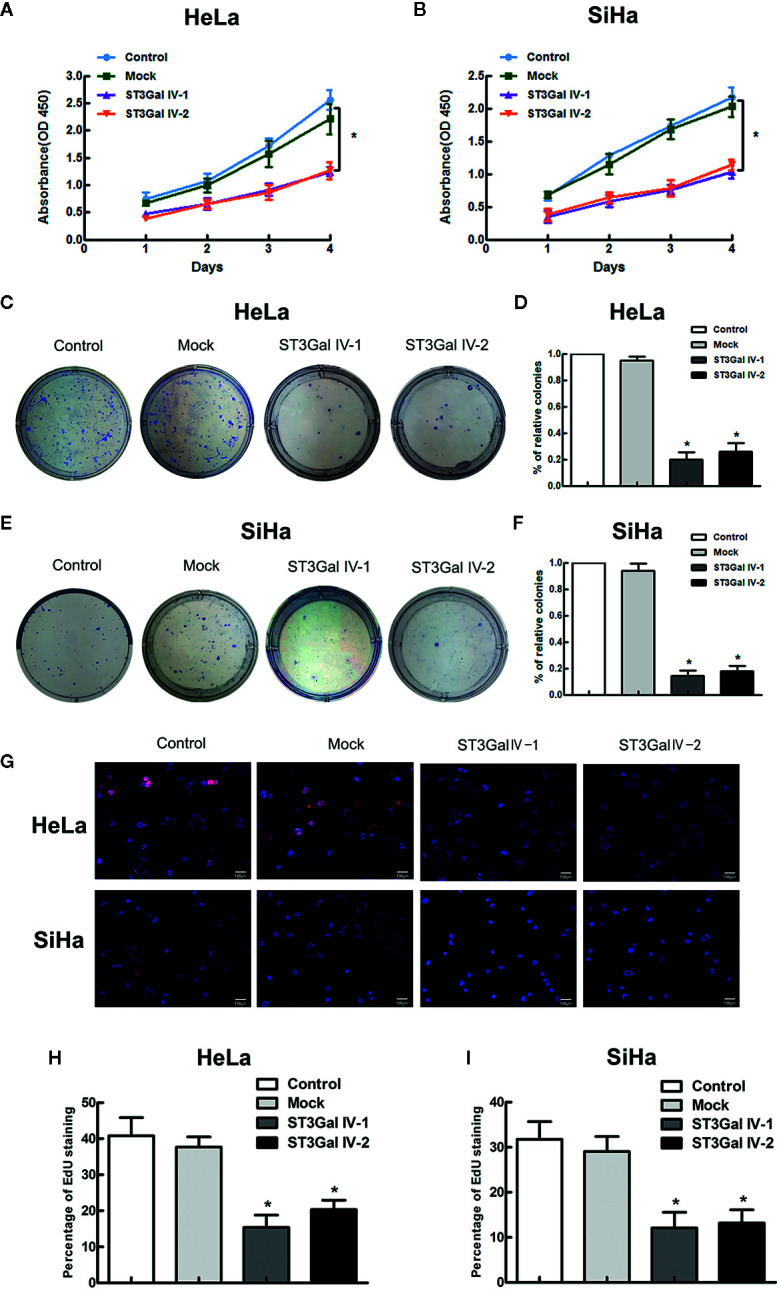
ST3Gal IV upregulation suppressed cervical cancer cell growth and colony formation *in vitro*. **(A, B)** The high expression of ST3Gal IV was connected with poor proliferation in HeLa and SiHa cell as shown by CCK-8 and performed in triplicate. **(C, D)** Cell colony formation assay adopted to further verify. **(E, F)** The relative cell colony formation rates of HeLa and SiHa cells from three independent experiments. **(G–I)** EdU proliferation assay revealed ST3Gal IV overexpression inhibited the DNA synthesis ability of HeLa and SiHa cells and data are from three independent experiments. (Magnification, ×20, scale bars, 100 μm). All values represent the mean ± SD (* *p* < 0.05).

To investigate the function of ST3Gal IV in the malignant phenotype of cervical cancer cells, CCK8, colony formation and EdU assays were performed to detect the cell proliferation and colony formation ability. CCK-8 results showed that overexpression of ST3Gal IV resulted in the inhibition of the growth rate of HeLa and SiHa cells, but there was no significant difference between the control group and the Mock group (**Figures 4A, B**). To further confirm the above results, cell proliferation was assessed by colony formation assay. Expectedly, ST3Gal IV overexpression decreased cell colony formation rate of HeLa (**Figures 4C, D**) and SiHa cells (**Figures 4E, F**). Furthermore, EdU assay showed that ST3Gal IV overexpression inhibited the DNA synthesis in HeLa and SiHa cells (**Figures 4G–I**) compared to the control groups. Together, our findings suggest ST3Gal IV overexpression has a negative effect on cell proliferation and colony formation ability of HeLa and SiHa cells *in vitro*.

### Upregulation of ST3Gal IV Blocks Cell Cycle in S Phase and Influences Notch1/p21/CDKs Signaling Pathway in Cervical Cancer Cells

**Figure 5:**
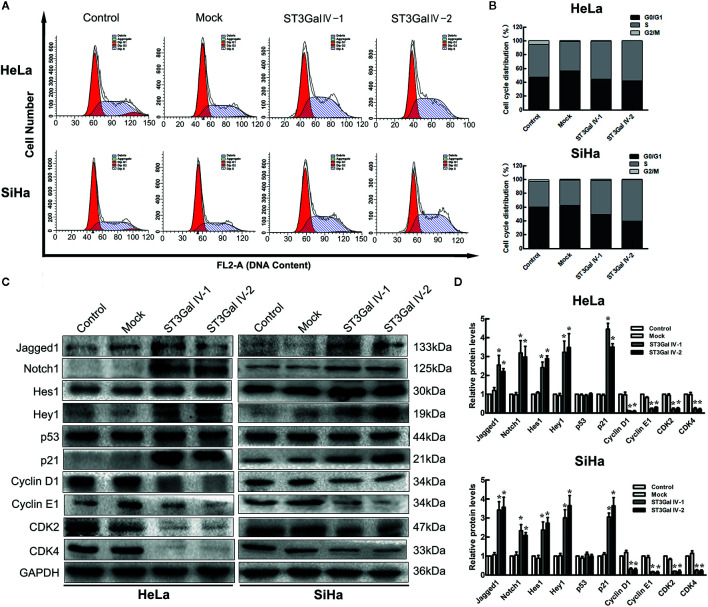
ST3Gal IV overexpression caused cell cycle arrest and influenced the Notch1/p21/CDKs signaling pathway in HeLa and SiHa cells. **(A, B)** Flow cytometry directly analyzed the cell cycle distribution of HeLa and SiHa cells. **(A)** Representative images showing the distribution of cells in G0/G1, S and G2/M phase. **(B)** Bar chart made from the data of three independent experiments. **(C)** Representative images of the main protein components of Notch/p21/CDKs signaling pathway measured by Western blot. **(D)** Protein expression levels were analyzed by Image Lab software. Data are from three independent experiments and represent the mean ± SD. * means *p* < 0.05.

To investigate the effects of ST3Gal IV upregulation on the proliferation of HeLa and SiHa cells, flow cytometry assay was performed to detect the cell cycle distribution. By calculating the number of cells in each cell cycle, we found that overexpression of ST3Gal IV in cervical cancer cells resulted in increased S phase cell populations and decreased G2/M cell populations, which was contrast with that in control groups (**Figures 5A, B**). This suggests that ST3Gal IV may mediate the proliferation of cervical cancer cells by affecting DNA synthesis. A large number of studies have revealed that Notch1 signaling pathway exerts great influence in tumor development. To elucidate whether this pathway was involved in ST3Gal IV-mediated cell proliferation, we analyzed the expression levels of related proteins in Notch1 signaling pathway. The results showed that the expression of Jagged1, Notch1, Hes1, Hey1 and p21 was significantly increased meanwhile CyclinD1, CyclinE1, CDK2, CDK4 expression levels were decreased observably in ST3Gal IV overexpressing cells compared to control cells (**Figures 5C, D**). However, there was no apparent change in the expression levels of p53 in ST3Gal IV overexpressing cells. These results indicate ST3Gal IV overexpression can induce cell cycle arrest in S phase and may affect cell proliferation through the Notch1/p21/CDKs signaling pathway.

### Notch Inhibitor DAPT Restores the Expression Levels of Proliferation-Related Proteins in ST3Gal IV Overexpression Cells

**Figure 6:**
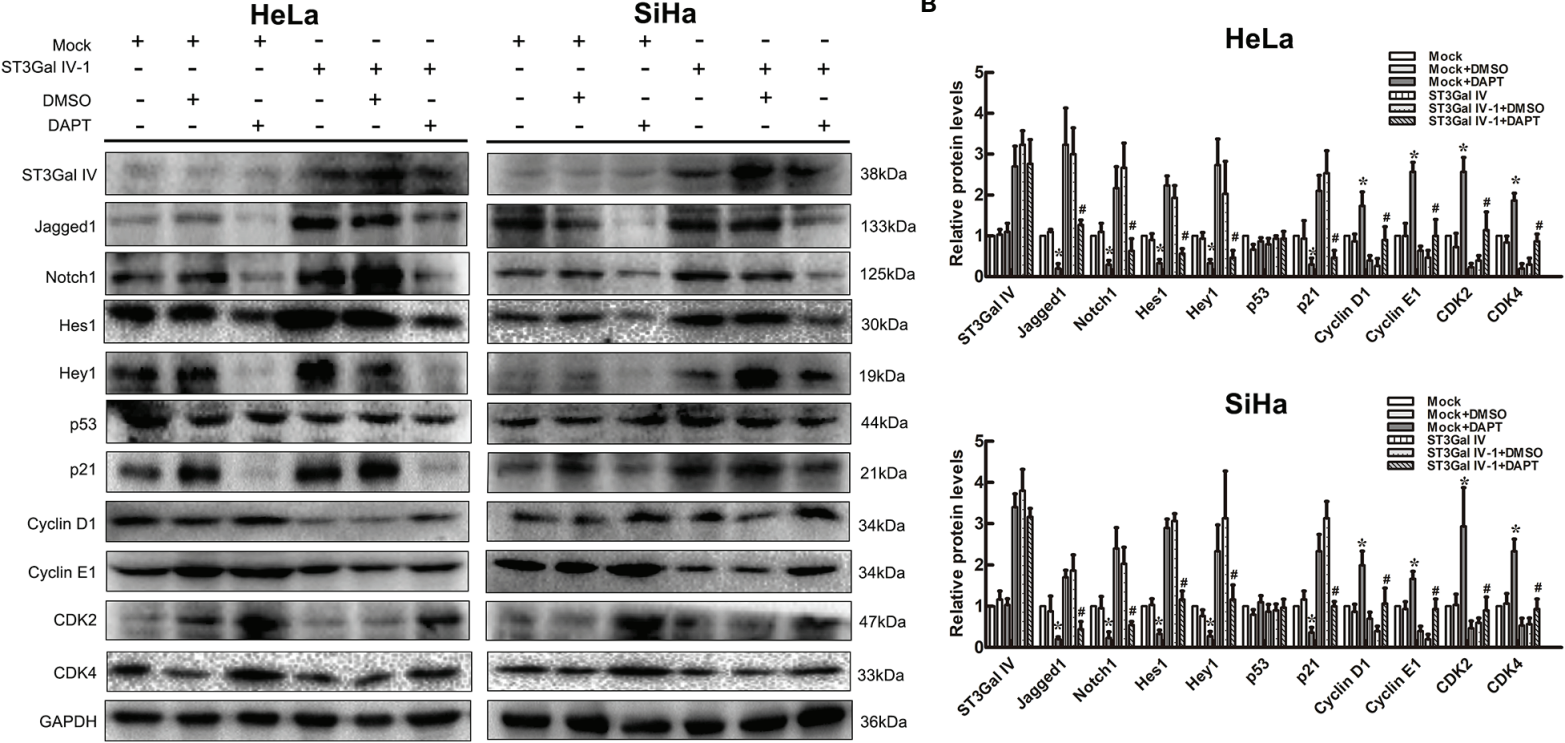
Notch inhibition increased ST3Gal IV upregulated induced cervical cancer cells proliferation. **(A)** Representative images showing protein expression of Notch/p21/CDKs signaling pathway. **(B)** Protein expression levels were analyzed by Image Lab software. All values are the mean ± SD of three independent experiments. *p < 0.05 compared to mock group cells with DMSO. ^#^ *p* < 0.05 compared to ST3Gal IV overexpression group cells with DMSO.

To further clarify that ST3Gal IV can mediate the growth and proliferation of cervical cancer cells through the Notch pathway, ST3Gal IV overexpressing cells and mock cells were treated with Notch1 inhibitor DAPT. As shown in **Figures 6A, B**, in mock/DAPT group, DAPT treatment significantly reduced the expression levels of Jagged1, Notch1, Hes1, Hey1, p21 and increased CyclinD1, CyclinE1, CDK2, CDK4 expression. However, there was no significant difference in p53 expression between the mock/DAPT group and ST3Gal IV/DAPT group. In addition, we found that the expression levels of the above-mentioned proteins in the ST3Gal IV/DAPT group were consistent with those in mock cells. This result suggests DAPT treatment can restore the expression levels of proliferation related proteins in ST3Gal IV upregulating cervical cancer cells. Taken together, these results confirm that ST3Gal IV overexpression inhibits the proliferation of cervical cancer cells *via* the Notch1/p21/CDKs signaling pathway.

### ST3Gal IV Overexpression Inhibits the Tumorigenicity of HeLa Cells in Nude Mice Through Notch1/p21/CDKs Pathway

**Figure 7:**
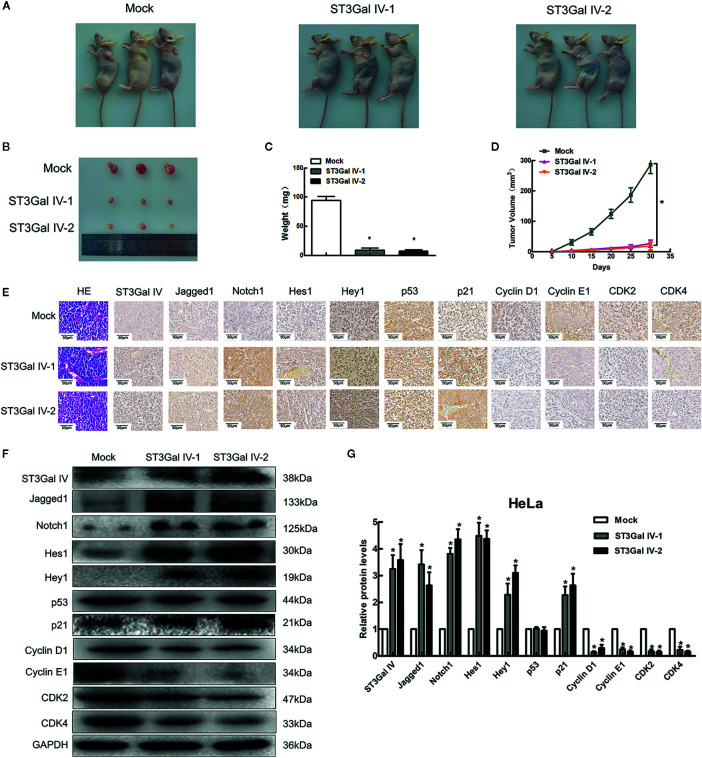
ST3Gal IV inhibited the cervical tumor formation in nude mice *via* Notch1/p21/CDKs. **(A, B)** Xenograft tumors derived from HeLa cells overexpressing ST3Gal IV or carrying a control vector after 30 days. **(C, D)** Average tumor weights were measured and tumor volumes were scaled every 5 days. **(E–G)** IHC and Western-blot detected the main protein components of Notch/p21/CDKs signaling pathway in tumor tissues. (Magnification, ×40, scale bars, 50 μm). **(F)** Representative images of Western blot are shown. Data are presented as mean ± SD of three independent experiments. * means *p* < 0.05.

To determine the effect of ST3Gal IV overexpression on the tumorigenic ability of cervical cancer cells *in vivo* subdermal injection of nude mice with HeLa cells overexpressing ST3Gal IV or carrying a control vector. The xenograft tumor size was dramatically decreased in the ST3Gal IV upregulation groups compared to mock group (**Figures 7A, B**). The average tumor weight and approximate tumor volume of the HeLa/ST3Gal IV tumors were significantly decreased compared with those in mock group (**Figures 7C, D**). Further, ST3Gal IV overexpression increased the Jagged1, Notch1, Hes1, Hey1 and p21 expression, meanwhile, decreased the expression levels of CyclinD1, CyclinE1, CDK2 and CDK4 in xenograft tumor tissues (**Figures 7E–G**). Therefore, these findings demonstrate that ST3Gal IV might be involved in the tumorigenesis and progression of cervical cancer *via* Notch1/p21/CDKs pathway *in vivo.*

## Discussion

Abnormal sialylation has been reported to impact tumor cell growth, differentiation, adhesion and invasion (19–21). In this study, we detected the expression level of ST3Gal IV in cervical cancer tissue microarray (n = 75), finding low ST3Gal IV expression in the cervical cancer tissues compared to normal cervix tissues. The results were consistent with those reported by Wang et al. that ST3Gal IV mRNA expression levels were downregulated in cervix squamous cell carcinoma (22). However, there is little research about the role of ST3Gal IV in cervical cancer progression. Subsequently we analyzed the correlation between ST3Gal IV expression and the pathological grade of cancer tissues, and statistical results showed that low expression of ST3Gal IV was dramatically related to the high malignant degree of cervical cancer tissues. Our findings indicated ST3Gal IV may act as a tumor suppressor in cervical cancer. Previous studies suggested that α2, 3 sialyltransferase and α2, 6 sialyltransferase compete with common substrate (8, 23). Consistent with these findings, the Lectin blot analysis showed that the overexpression of ST3Gal IV can decrease expression of α2, 3-linked sialic acid in cervical cancer cells. However, how the decrease of α2, 6 sialylation affects the growth and proliferation of cervical cancer cells is what we need to explore in the next research.

The results of Mass Spectrometry suggested that high mannose is the main *N*-glycan components in any kind of cell lines we used, which is consistent with the *N*-glycosylation characteristics of cell membrane proteins. Although HeLa and SiHa are both cervical cancer cell lines, the different origins lead to slight differences in glycan pattern between them, which is mainly reflected in the different glycan pattern between peak 16 and 45. After the overexpression of ST3Gal IV, the glycan pattern of these two cell lines have changed observably, especially the signal intensity of the four polysaccharides of peak 29,36,40,45 increased significantly. The results indicated the transfection of ST3Gal IV is successful since the four polysaccharides contained sialic acid residues. Although our experimental results could not determine the linkage between sialic acid residues and glycan is α2, 3-, or α2, 6-linkage, these sialylated polysaccharides increased significantly with overexpression of ST3Gal IV. Therefore, we speculate that α2, 3-sialylated glycan are increased due to the overexpression of ST3Gal IV in the process of 2, 3 sialyltransferases competing with α2, 6 sialyltransferases for common substrate. In addition, we also found a very interesting phenomenon in the mass spectrometry analysis of HeLa/ST3Gal IV cell line. The percentage of peak 2 [*m/z* = 1590.67, (HexNAc)_1_(Fuc)_1_ + (Man)_3_(GlcNAc)_2_], peak 7 [*m/z* = 1835.89, (HexNAc)_2_(Fuc)_1_ + (Man)_3_(GlcNAc)_2_], peak 13 [*m/z* = 2081.14, (HexNAc)_3_(Fuc)_1_ + (Man)_3_(GlcNAc)_2_] expression in total glycan increased significantly, and the results of repeated experiments were consistent with this. However, this phenomenon was not found in SiHa/ST3Gal IV cell line and the specific reason is not clear and needs further study.

It has been reported that ST3Gal IV is up-regulated and plays a pro-cancer role in numerous cancers including pancreatic adenocarcinoma, gastric cancer and leukemia (11, 24–26). However, our study found that upregulation of ST3Gal IV inhibited cervical cancer cell proliferation *in vitro* and mediated cell cycle arrest in S phase. Furthermore, the *in vivo* xenograft experiment also suggested that HeLa cells with high ST3Gal IV expression showed lower tumorigenicity rates and growth than that in mock group. We speculate that this may be caused by epigenetic changes, such as DNA hypermethylation. As Kawamura YI reported that the hypermethylation of ST3Gal VI gene induces the aberrant glycosylation and expression of carbohydrate antigens by silencing of the activity of glycosyltransferases in gastrointestinal cancer (27). Therefore, whether ST3Gal IV has undergone the epigenetic changes in cervical cancer cells still needs to be explored in our future work. In addition, the difference between subcutaneous microenvironment and cervical environment in nude mice is one of the limitations of *in vivo* experiment. Another limitation is the presence of *N*-acetylneuraminic acid (Neu5Ac) and *N*-glycolylneuraminic acid (Neu5Gc) in nude mice, while humans only contain Neu5Ac because of a mutation in CMP-sialic acid hydroxylase (28). These make some errors in our experiment and we look forward to building a more rigorous xenograft model in our next experiment.

Recent findings have revealed a variety of molecular events involved in cervical carcinogenesis, such as Wnt signaling, C-Met/HGF signaling pathway, PI3K/AKT/mTOR signaling pathway, etc. (29–31). Notch signaling was involved in cell proliferation, differentiation and apoptosis as a mechanism for evolutionarily conserved intercellular interaction. The overexpression of Notch1 signaling proteins in cervical cancer suggested that Notch1 may promote tumor progression (32–34). However, the effect of Notch signaling on cervical cancer is not entirely clear. Other studies demonstrated that the activation of Notch signaling displayed the suppression on cancer cells proliferation (35, 36). In our study, ST3Gal IV overexpression induced the up-regulation of Notch1, the cognate ligand, Jagged1 and the Notch downstream targets Hes1 and Hey1. The tumor suppressor p21, one of the Notch1 target genes (37), was also increased in cervical cancer cells overexpressed by ST3Gal IV. Furthermore, consistent with the fact that p21 is a cyclin-dependent kinase inhibitor, we found that levels of cyclin-related proteins such as Cyclin D1, Cyclin E1, CDK2 and CDK4 were reduced in cervical cancer cells overexpressed by ST3Gal IV. However, we did not observe a significant change in p53 protein level. The use of Notch1 inhibitor DAPT further confirmed our results that ST3Gal IV can regulate Notch1/p21/CDKs signaling pathway. Therefore, these observations clearly suggest that ST3Gal IV mediates the growth and proliferation of cervical cancer cells *via* the Notch/p21/CDKs pathway.

Notch protein undergoes post-translational modification to accomplish its activity and functions, and glycosylation is a significant factor regulating Notch signaling pathway (38–40). Several extracellular epidermal growth factor-like (EGF) domains of Notch are modified with *O-*fucose and *O-*glucose glycans as well as *N*-glycans that alter receptor sensitivity to ligand stimulation (38, 41). These studies indicated that *N-*glycosylation and *O-*glycosylation played essential roles in Notch1 signaling pathway. Our result revealed that ST3Gal IV overexpression caused a significant increase in the expression of levels of Jagged1, Notch1 and the downstream molecules, and the upregulation of p21 inhibited the expression of CDKs, suggesting that ST3Gal IV might participate in the modulation of Notch receptors or ligands in cervical cancer cells. There are many crosstalks between Notch signals and other signaling pathways (such as TGF-β/Smad, Wnt, NF-κB, PI3K/Akt, etc.) (42–46). Therefore, further studies are needed to elucidate the precise molecular mechanisms by which ST3Gal IV modulates the Notch1/p21/CDKs signaling pathway in cervical cancer.

Taken together, our research indicated that ST3Gal IV was negatively associated with the pathological grade of cervical cancer tissues, and the overexpression of ST3Gal IV inhibited the growth and proliferation of cervical cancer cells *in vivo* and *in vitro* through Notch1/p21/CDKs signaling pathway. Therefore, ST3Gal IV might be a potential target for the prognosis determination and treatment of cervical cancer in the future.

## Data Availability Statement

The datasets generated for this study are available on request to the corresponding authors.

## Ethics Statement

All care and experimental procedures were performed in accordance with the guidelines approved by the Dalian Medical University Animal Ethics Committee (Animal protocol No. AEE18067). In addition, all animal experiments in the present study were consistent with the National Institutes of Health guide for the care and use of laboratory animals.

## Author Contributions

SJW, XS, YSW, and XC designed the study. YSW, XC, and WD contributed equally to this study and performed all experiments. WD and YW completed the mass spectrometry analysis experiment. WD, YW, and GL analyzed the mass spectrometry analysis data. YJ, ZX, CX, and LZ participated in cell culture and cell transfection experiments. AW analyzed the association between ST3Gal IV expression and clinical data. XY, XS, and SDW participated in the tissues detection. SJW, YSW, and XC wrote and revised the manuscript. All authors contributed to the article and approved the submitted version.

## Funding

This work is supported by Natural Science Foundation of Liaoning Province and Transverse Program of Dalian Medical University (2019-ZD-0640, 505526) and the Liaoning Provincial Program for Top Discipline of Basic Medical Sciences.

## Conflict of Interest

The authors declare that the research was conducted in the absence of any commercial or financial relationships that could be construed as a potential conflict of interest.
