# Supplementary material for: ST3Gal IV Mediates the Growth and Proliferation of Cervical Cancer Cells In Vitro and In Vivo Via the Notch/p21/CDKs Pathway
Source: Front Oncol. 2021 Feb 1;10:540332. doi: 10.3389/fonc.2020.540332 (PMC7882721; doi:10.3389/fonc.2020.540332)
Supplement: Supplementary file 1 [file Table_1.docx]

**Supplementary information**

Supplemental Table S1. Information of ST3Gal IV overexpression vector.

| Gene | ST3GAL4 |
| --- | --- |
| Accession number | NM_001254757.2 |
| Cloning vector | pcDNA3.1(-) |
| Insert size | 1002bp (CDS region: 218-1219) |
| Insert sequence | ATGGTCAGCAAGTCCCGCTGGAAGCTCCTGGCCATGTTGG 40  CTCTGGTCCTGGTCGTCATGGTGTGGTATTCCATCTCCCG 80  GGAAGACAGGTACATCGAGCTTTTTTATTTTCCCATCCCA 120  GAGAAGAAGGAGCCGTGCCTCCAGGGTGAGGCAGAGAGCA 160  AGGCCTCTAAGCTCTTTGGCAACTACTCCCGGGATCAGCC 200  CATCTTCCTGCGGCTTGAGGATTATTTCTGGGTCAAGACG 240  CCATCTGCTTACGAGCTGCCCTATGGGACCAAGGGGAGTG 280  AGGATCTGCTCCTCCGGGTGCTAGCCATCACCAGCTCCTC 320  CATCCCCAAGAACATCCAGAGCCTCAGGTGCCGCCGCTG 360  TGTGGTCGTGGGGAACGGGCACCGGCTGCGGAACAGCTCAC 400  TGGGAGATGCCATCAACAAGTACGATGTGGTCATCAGATT 440  GAACAATGCCCCAGTGGCTGGCTATGAGGGTGACGTGGGC 480  TCCAAGACCACCATGCGTCTCTTCTACCCTGAATCTGCCC 520  ACTTCGACCCCAAAGTAGAAAACAACCCAGACACACTCCT 560  CGTCCTGGTAGCTTTCAAGGCAATGGACTTCCACTGGATT 600  GAGACCATCCTGAGTGATAAGAAGCGGGTGCGAAAGGGTT 640  TCTGGAAACAGCCTCCCCTCATCTGGGATGTCAATCCTAA 680  ACAGATTCGGATTCTCAACCCCTTCTTCATGGAGATTGCA 720  GCTGACAAACTGCTGAGCCTGCCAATGCAACAGCCACGGA 760  AGATTAAGCAGAAGCCCACCACGGGCCTGTTGGCCATCAC 800  GCTGGCCCTCCACCTCTGTGACTTGGTGCACATTGCCGGC 840  TTTGGCTACCCAGACGCCTACAACAAGAAGCAGACCATTC 880  ACTACTATGAGCAGATCACGCTCAAGTCCATGGCGGGGTC 920  AGGCCATAATGTCTCCCAAGAGGCCCTGGCCATTAAGCGG 960  ATGCTGGAGATGGGAGCTATCAAGAACCTCACGTCCTTCT 1000  GA 1002 |
